# Metagenomic next-generation sequencing for the clinical identification of spinal infection-associated pathogens

**DOI:** 10.3389/fcimb.2024.1437665

**Published:** 2025-01-07

**Authors:** Tengfei Shi, Yuhan Lin, Xuexin Zheng, Hongliang Ruan, Rui Zhang, Yinhuan Liu, Shaohan Xu, Huafeng Wang

**Affiliations:** ^1^ Department of Clinical Laboratory, Fuzhou Second General Hospital, Fuzhou, Fujian, China; ^2^ Department of Spine Surgery, Fuzhou Second General Hospital, Fuzhou, Fujian, China; ^3^ Department of Clinical Laboratory, Fuzhou Second General Hospital (Fuzhou Maternal and Child Health Hospital), Fuzhou, Fujian, China

**Keywords:** metagenomic next-generation sequencing, microbial culture, spinal infection, pathogens, sensitivity, specificity

## Abstract

**Background:**

This study aimed to evaluate the efficacy of metagenomic next-generation sequencing (mNGS) technology for identifying pathogens associated with spinal infection (SI).

**Methods:**

A retrospective analysis was conducted on clinical data from 193 patients with suspected SI between August 2020 and September 2024. Based on histopathological results, the patients were divided into the SI group (n=162) and the non-SI group (n=31). The diagnostic performance of mNGS technology was compared with that of laboratory examination, imaging examination, and microbial culture.

**Results:**

Among SI group, mNGS detected 135 pathogens in 77.78% (126/162) of the cases, including nine cases of multiple infections. One or more pathogens were detected using mNGS in 86 patients with SI and negative microbial cultures. *Staphylococcus aureus* (22.22%, n=30) and *Mycobacterium tuberculosis* (22.22%, n=30) were the major pathogens, while various rare pathogens such as anaerobes, *Brucella*, and *Coxiella burnetii* were also detected. For the 40 cases with positive results for both culture- and mNGS-based identification, high consistency (77.50%) was observed. Antibiotic use did not significantly affect the mNGS detection rate (P = 0.45). There was no significant difference in the positivity rate of mNGS between CT-guided needle biopsy (80.00%) and surgical sampling (77.17%) (P = 0.72). The sensitivity of mNGS (77.78%) was significantly higher than that of traditional microbial culture (27.16%), and the specificity was similar (90.32% vs. 96.77%). Although the sensitivities of erythrocyte sedimentation rate-based assay (91.36%), magnetic resonance imaging (88.27%), and C-reactive protein-based assay (87.65%) were better than those of mNGS, their specificities were generally low (20%-40%).

**Conclusion:**

The pathogens responsible for SI are complex and diverse. As a novel diagnostic method, mNGS exhibits a high sensitivity and extensive pathogen coverage for SI diagnosis. When combined with imaging and laboratory indicators, mNGS can significantly improve the accuracy of SI diagnosis and provide strong support for clinical treatment.

## 1 Introduction

Spinal infection (SI) are primarily caused by bacteria, fungi, and other pathogens that invade the spinal bone and soft tissue via the blood circulation or direct attack (Gregori et al., 2019). SI can be divided into pyogenic infections (bacteria) and granulomatosis infections (fungi, parasites, etc.), with significant differences in antibiotic selection and treatment strategies (Lener et al., 2018; Kleck et al., 2024). Therefore, the rapid and accurate identification of pathogens is essential for the treatment of SI.

Traditional microbial culture, the gold standard for infection diagnosis, plays a pivotal role in the identification of SI pathogens; however, it is time-consuming and has a low positivity rate (only approximately 20% to 50%) (Guo et al., 2022; Li et al., 2024; Lin et al., 2023). Molecular diagnostic techniques, such as multiplex polymerase chain reaction (PCR) and 16S rRNA gene sequencing, have narrow pathogen detection coverage, are unable to identify multiple microbial infections, and may miss uncommon pathogens. Metagenomic next-generation sequencing (mNGS), an emerging molecular diagnostic method, has attracted considerable clinical attention. mNGS allows direct pathogen detection from biological samples without prior assumptions or reliance on traditional cultures. It can be used to simultaneously analyze thousands of DNA fragments, enabling the comprehensive detection of pathogens, such as bacteria, fungi, viruses, and parasites (Gu et al., 2019). This technology has been applied to the detection of pathogens in various infectious diseases, such as bloodstream, nervous, urinary, and respiratory systems, and has shown great potential in orthopedic infections (Yin et al., 2022; Wilson et al., 2019; Jia et al., 2023; Guo et al., 2021; Huang et al., 2020). However, instances of clinical utilization with mNGS for SI remains relatively limited, with only a few relevant reports.

Therefore, this study aimed to comprehensively evaluate the diagnostic performance and clinical application of mNGS for the detection of SI pathogens. This is expected to provide clinicians with a more accurate, rapid, and comprehensive diagnostic tool to improve the treatment and management of patients with SI.

## 2 Materials and methods

### 2.1 Study design

A retrospective approach was adopted to collect clinical data of patients with suspected SI from the Fuzhou Second General Hospital from August 2020 to September 2024. The data included demographic characteristics, imaging results, laboratory findings, histopathological results, microbial cultures, and mNGS test results. Cases were categorized into the SI group and the non-SI group based on the histopathological results to investigate the clinical characteristics of patients with SI, and compare the diagnostic efficacy of mNGS with laboratory and imaging examinations. This study was approved by the Ethics Committee of the Fuzhou Second General Hospital (No. 2024200), and all patients provided written informed consent.

### 2.2 Inclusion and exclusion criteria

Inclusion criteria: ① Patients with Clinical Suspicion of SI: Suspected SI is defined as the presence of new or worsening spinal pain and/or neurological symptoms, accompanied by at least one of the following abnormalities: fever; elevated erythrocyte sedimentation rate (ESR) or C-reactive protein (CRP) levels; bloodstream infection or infective endocarditis. Radiological findings should be consistent with the characteristics of discitis, spondylitis, or spondylodiscitis, which include vertebral endplate destruction, disc inflammation, and the presence of necrosis or pus within the intervertebral disc space, paraspinal soft tissue, or epidural space (Berbari et al., 2015; Huang et al., 2023; Yin et al., 2024). ② Patients underwent mNGS, traditional microbial cultures, inflammatory marker tests including CRP, ESR, and procalcitonin (PCT), as well as imaging tests including computed tomography (CT) and magnetic resonance imaging (MRI). ③ The final diagnosis of spinal infection is based on pathological examination. The criteria for a positive diagnosis include nodular granulomatous inflammation, suppurative inflammation, or the presence of infectious lesions on pathological examination (Huang et al., 2023).

Exclusion criteria: ① Patients whose mNGS results indicated potential contamination during sample collection, transportation, or processing were excluded. Potential contamination is defined as follows: sampling not performed or supervised by a spinal surgeon, non-aseptic sampling procedures, samples not collected from a sealed body cavity or having any contact with skin, and samples not stored under sealed conditions or transported via an unqualified cold chain during the collection and transportation process. ② Patients with incomplete clinical data; ③ Patients who declined mNGS testing; and ④ Patients who were ultimately diagnosed with spinal tumors.

### 2.3 Sample collection and processing

Tissue samples were collected using CT-guided needle biopsy or open surgery. Fresh samples were immediately divided into three portions: one portion was sent to the laboratory for microbial culture, another portion was fixed in formalin for histopathological analysis in the pathology department, and the remaining portion was immediately placed in sterile, nuclease-free, or other amplification inhibitor-free, special sealed containers, preserved using dry ice, and transported under cold-chain conditions to the Agene Genomics Laboratory (Fuzhou, China), where it underwent mNGS testing within 24 hours.

### 2.4 Culture procedure

Tissue samples were added to 5 mL brain-heart infusion broth, processed using a vortex mixer and grinding machine (Shanghai Jingxin Industrial Development Co., Ltd., Shanghai, China) and inoculated onto blood agar plates for microbial culture under anaerobic and aerobic conditions. The culture period is usually 7 days but may be extended to 14 days in special circumstances, especially in the presence of negative cultures with a high clinical suspicion of SI. When colonies grew on the blood agar plates, individual colonies were picked, and the isolated strains were further verified and identified using MALDI-TOF MS (Bruker Daltonics GmbH, Billerica, MA, USA) and Phoenix 100 (Becton Dickinson and Company, Sparks, MD, USA).

### 2.5 mNGS procedure

mNGS testing followed a standard protocol, including sample processing, DNA extraction, library construction and sequencing, and bioinformatics analysis. Specifically: a 3×3×3 mm³ tissue cube or a 0.5 cm biopsy tissue is obtained from the patient using a disposable blade and placed in 300 µL of preservative solution. Tissue digestion buffer, lysis buffer, and buffer solution are added to a grinding tube containing grinding beads, and after a 10 minute cell disruption process, DNA is extracted using a magnetic bead-based pathogenic microorganism DNA extraction kit (Fuzhou OJX Biotechnology Co., Ltd, Fuzhou, China). The DNA concentration is measured using a fluorometer (Qubit 4.0, Invitrogen). A DNA sample library preparation kit (Fuzhou OJX Biotechnology Co., Ltd, Fuzhou, China) is utilized for library preparation, followed by the circularization of double-stranded libraries using a DNA cyclization reaction kit (Fuzhou OJX Biotechnology Co., Ltd, Fuzhou, China) to prepare DNA Nanoballs. The DNA Nanoball concentration is verified again using a Qubit 4.0 fluorometer to ensure a concentration of ≥8 ng/μL.

Sequencing is performed on the MGISEQ-200 platform (MGI Tech Co., Ltd., Shenzhen, China) using the MGISEQ-200RS high-throughput sequencing reagent kit (MGI Tech Co., Ltd., Shenzhen, China) in SE50 mode. After splitting the sequencing data, filtering out low-quality reads, and removing adapters, the data is aligned with the human genome (hg38+NCBI partial) using bwa-mem2 (v2.1) to exclude human DNA. Unaligned sequences are extracted using samtools (1.16.1) fasta -f 4.

PCR duplicates are further removed using seqkit v0.11.0, and the remaining sequences are aligned with the Kraken 2 Standard (kraken2 2.0.7-beta) and NCBI NT databases (blastn v2.9.0+). Each round of mNGS testing includes both a negative control (composed of plasma-free nucleic acids and fragmented human genomic DNA) and a positive control (a mixture containing inactivated Klebsiella pneumoniae, Streptococcus pneumoniae, Mycobacterium tuberculosis, and human cytomegalovirus).

The positivity criteria were as follows (Wilson et al., 2019; Schlaberg et al., 2017; Luan et al., 2021): (1) Sequence data met quality control standards, with library concentration above 50 pM, Q20 value greater than 85%, and Q30 value greater than 80%; (2) No target species were detected in the negative control on the same chip, or reads per million (sample)/reads per million (NC) ≥ 5; (3) Bacterial diagnostic threshold: genus relative abundance > 15% and sequence count > 30; (4) Fungal diagnostic threshold: genus relative abundance > 15% and sequence count > 50; (5) For the pathogens of high clinical concern and difficult to detect such as *M. tuberculosis* and *Brucella*, the detection of one specific sequence could be judged as positive (Expert Group on Consensus for High-Throughput Sequencing, 2023; Chinese Society of Laboratory Medicine, 2020);

The laboratory procedures and bioinformatics analyses for mNGS were conducted by Agene Genomics Laboratory (Fuzhou, China). All results were reviewed by at least two experienced clinicians, one laboratory microbiologist and one bioinformatics expert to distinguish between infection, colonization, and contamination. The review process considered factors such as sample type, testing history, clinically relevant pathogens, microbial pathogenicity, and clinical medication information. When determining whether an opportunistic pathogen is the causative agent, consideration should be given to the patient’s immune status, underlying diseases, and the source of the specimen. In the presence of a large number of background or miscellaneous bacterial sequences without a dominant microorganism, contamination should be the primary consideration, followed by the possibility of an opportunistic pathogen.

### 2.6 Statistical analysis

An exhaustive statistical analysis was performed on all collected data, including the patients’ clinical characteristics and pathogen detection results. Descriptive data are presented as mean (standard deviation, SD) and median (interquartile range, IQR), and categorical variables are presented as frequencies and percentages. To compare the performances of the different detection methods, statistical methods such as the unpaired *t*-test, Mann–Whitney U test, chi-square test, and Fisher’s exact test were used. All statistical analyses were performed using GraphPad Prism 9.5 software, with the significance level set at 0.05.

## 3 Results

### 3.1 Demographic characteristics

Based on the inclusion and exclusion criteria, a total of 193 patients with suspected SI were divided into the SI group (n=162) and the non-SI group (n=31) according to histopathological results. Among SI group, 86 were male and 76 were female, with an median age of 66 years (IQR: 15 years). The most common site of infection was the lumbar spine (131 patients, 80.86%) followed by the thoracic spine (30 patients, 18.52%). As the procalcitonin (PCT) detection threshold was set at 0.05, values below this threshold were considered 0.025 for quantitative statistical analysis, according to the literature (Cheng et al., 2023). There were significant differences in ESR, CRP and MRI findings between the SI group and the non-SI group. Further details are listed in **Table 1**. The clinical data of all patients, as well as the relative abundance and sequence counts of the pathogens, are available for review in the **Supplementary Materials**.

**Table 1:** Demographic and clinical characteristics.

Characteristic	SI group (n=162)	Non-SI group (n=31)	P value
**Age, years, Median (IQR)**	66 (15)	67 (16)	0.44
Sex, n (%)			
Male	86 (53.09)	20 (64.52)	0.24
Female	76 (46.91)	11 (35.48)	0.24
Infection site, n (%)			
Cervical spine	1 (0.62)	0 (0.00)	>0.99
Thoracic spine	30 (18.52)	8 (25.81)	0.35
Lumbar spine	131 (80.86)	22 (70.97)	0.21
Sacral vertebrae	0 (0.00)	1 (3.22)	0.16
Laboratory findings			
CRP, mg/L, Median (IQR)	33.98 (56.25)	20.70 (50.89)	0.00
ESR, mm/h, Median (IQR)	68.50 (49.50)	53.45 (33.61)	0.01
PCT, (ng/ml), Median (IQR)	0.07 (0.15)	0.06 (0.10)	0.91
**CT, n (%)**	119 (73.46)	23 (74.19)	0.93
**MRI, n (%)**	143 (88.27)	23 (74.19)	0.04
Underling disease, n (%)			
Diabetes	34 (20.99)	6 (19.35)	0.84
Hypertension	61 (37.65)	11 (35.48)	0.82
**Operation history, n (%)**	90 (55.56)	21 (67.74)	0.21

SI, spinal infection; IQR, interquartile range; CRP, c-reactive protein; ESR, erythrocytesedimentation rate; PCT, procalcitonin; CT, computed tomography; MRI, magnetic resonance imaging.

### 3.2 Results of microbial culture and mNGS

**Figure 1:**
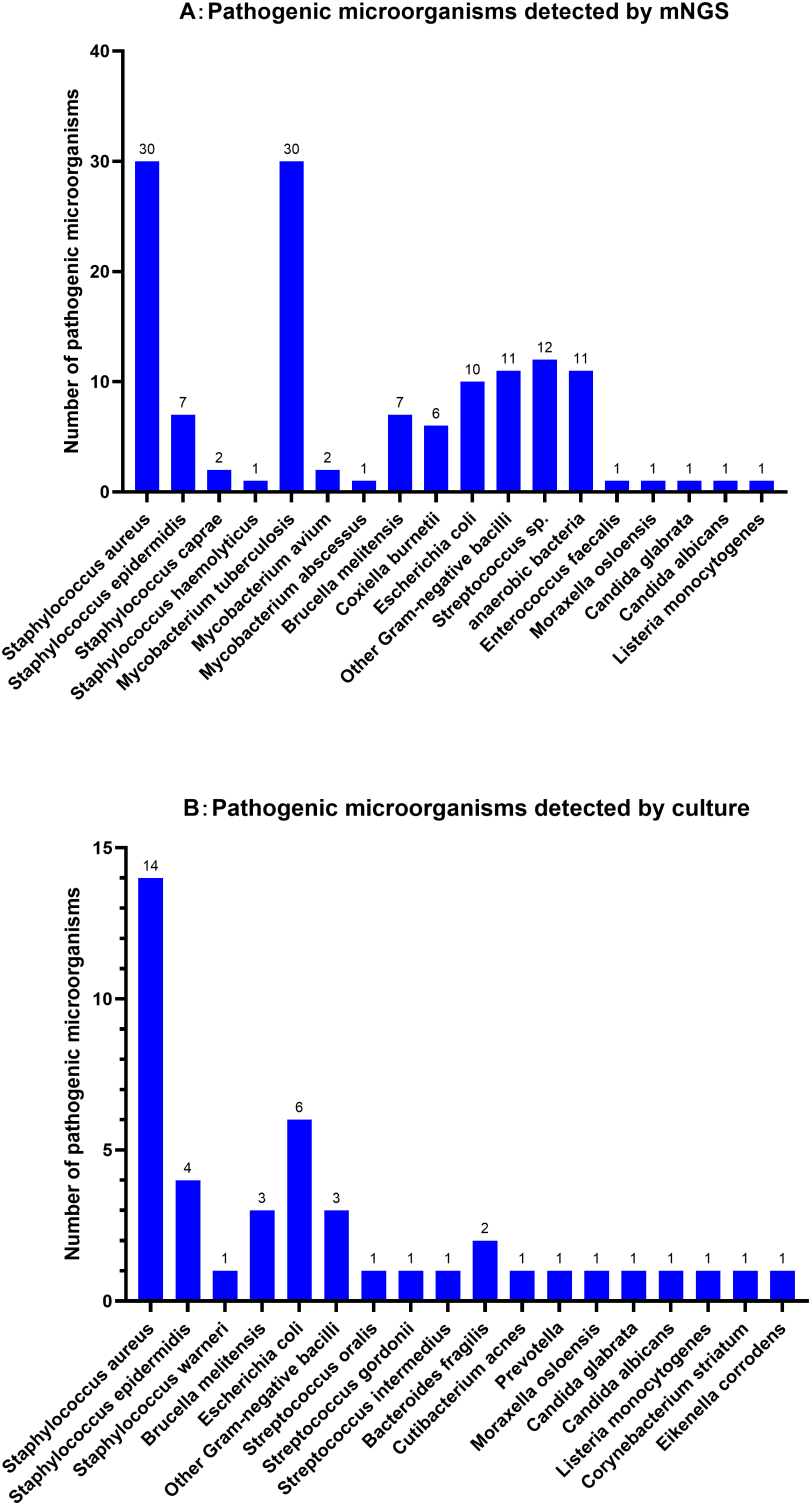
Distribution of pathogen detected by mNGS **(A)** and microbial Culture **(B)** for Spinal infection.

In the SI group, a total of 44 species of pathogens were detected through microbial cultures, including *Staphylococcus aureus* (31.82%, n=14), *Escherichia coli* (13.64%, n=6), *Staphylococcus epidermidis* (9.09%, n=4), and *Brucella melitensis* (6.82%, n=3). The positive detection rate among the tested samples was 27.16% (44/162). In contrast, mNGS detected pathogens in 77.78% (126/162) of the samples, identifying 135 pathogens, in nine patients infected with multiple pathogens. mNGS also detected one or more pathogens in 86 patients with SI and negative culture results. The main pathogens identified were *S. aureus* (22.22%, n=30), *M. tuberculosis* (22.22%, n=30), *Streptococcus* spp. (8.89%, n=12), anaerobes (8.15%, n=11), *Escherichia coli* (7.41%, n=10), and *B. melitensis* (5.19%, n=7). Most *Streptococcus* spp. belonged to the normal microbiota of the oral cavity, including *Streptococcus mitis*, *Streptococcus oralis* and *Streptococcus gordonii*. *Coxiella burnetii*, which is rarely reported in literature, was detected in six samples. However, four culture-positive cases were missed during mNGS detection; these were associated with *S. aureus* (n=1), *S. epidermidis* (n=2) and *Moraxella osloensis* (n=1). Further details are shown in **Figure 1**.

**Figure 2:**
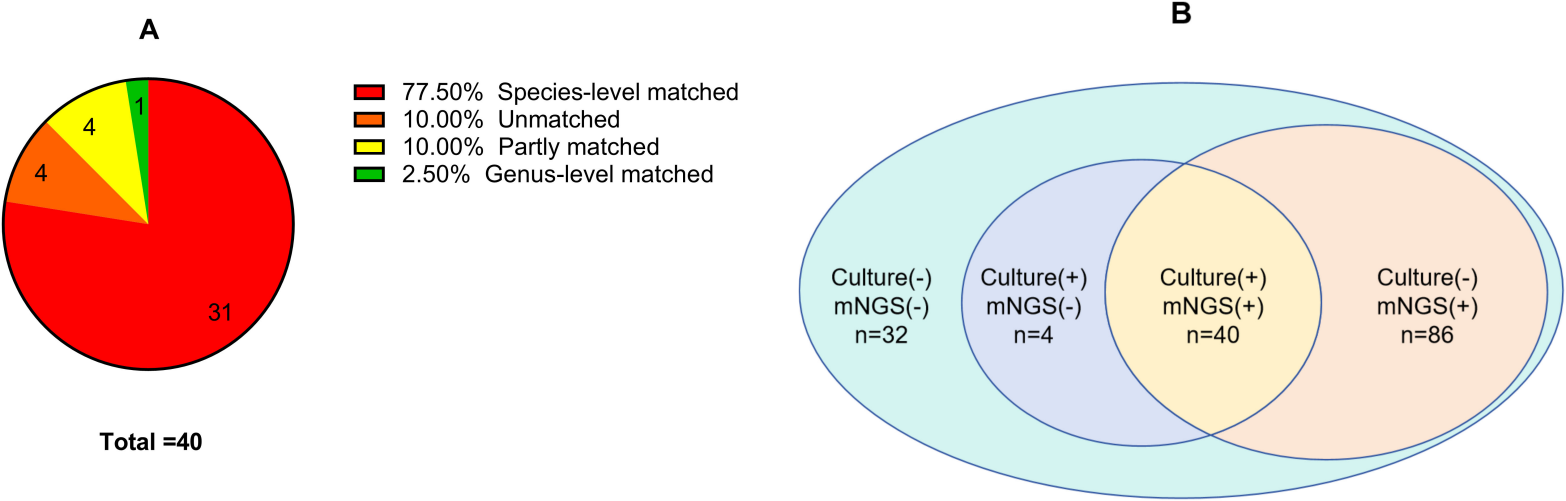
**(A)** Concordance between metagenomic next-generation sequencing and culture positivity in spinal infection; **(B)** To compare the number of pathogens detected by metagenomic next-generation sequencing and culture in spinal infection.

Among the 40 SI cases with positive results for both culture and mNGS, 31(77.50%) showed complete consistency in species-level identification, one (2.50%) showed consistency at the genus level, four (10.00%) showed partial consistency, and four (10.00%) showed complete disagreement. Further details are shown in **Figure 2**.

### 3.3 Impact of antibiotic use on mNGS

The 162 patients with SI were divided into two groups based on the use of antibiotics before sampling: an antibiotic use group (118 cases) and a non-use group (44 cases). The main antibiotics used were vancomycin (74.58%, n=88), cefoperazone/sulbactam (5.08%, n=6), and levofloxacin (4.24%, n=5). The results showed that the detection rate of mNGS in the antibiotic-use group was 76.27% (90/118), whereas that in the non-use group was 81.82% (36/44), with no significant difference between the two groups (P = 0.45).

### 3.4 Impact of sampling method on mNGS

Based on the differences in sampling methods, 162 patients with SI were classified into the needle biopsy group (n=35) and the open surgery group (n=127). The mNGS detection rates in the CT-guided needle biopsy and surgical sampling groups were 80.00% (28/35) and 77.17% (98/127), respectively. Statistical analysis revealed no significant difference between the two methods (P = 0.72). However, the impact of sampling method on culture results was significant, with a detection rate of only 11.43% (4/35) in the needle biopsy group, significantly lower than the 31.50% (40/127) in the surgical sampling group, indicating a statistically significant difference (P = 0.02). Further details are presented in **Table 2**.

**Table 2:** Comparison of detection rates between mNGS and culture using different sample collection methods.

Methods	needle biopsy group (n=35)	Open surgery group (n=127)	P value
mNGS, n (%)	28 (80.00)	98 (77.17)	0.72
Culture, n (%)	4 (11.43)	40 (31.50)	0.02

mNGS, metagenomic next-generation sequencing.

### 3.5 Diagnostic performance of mNGS in SI

We compared the diagnostic efficacy of mNGS with traditional microbial culture, laboratory tests, and imaging studies in detecting SI. We defined the normal ranges for laboratory indicators as follows: ESR (15 mm/h for males, 20 mm/h for females), CRP (8 mg/L), PCT (0.05 ng/mL), and values above these ranges were considered positive. Positive CT and MRI results indicate the presence of inflammation or infection. Statistical analysis showed that the sensitivity, specificity, positive predictive value, negative predictive value and accuracy of mNGS were 77.78%, 90.32%, 97.67%, 43.75% and 79.79%, respectively. The sensitivity and specificity of traditional microbial culture were 27.16% and 96.77%, respectively. These results indicate that mNGS has higher sensitivity and similar specificity in the diagnosis of SI. The sensitivity of MRI (88.27%), CRP (87.65%) and ESR (91.36%) was higher than that of mNGS, but the specificity was generally low (about 20%-40%). Further details are presented in **Table 3**.

**Table 3:** Comparison of the diagnostic value of mNGS and other methods (%) (n=162).

Methods	Sensitivity	Specificity	PPV	NPV	Accuracy
mNGS	77.78	90.32	97.67	43.75	79.79
Culture	27.16	96.77	97.78	20.27	38.34
CT	73.46	25.81	83.80	15.69	65.80
MRI	88.27	25.81	86.14	29.63	78.24
CRP	87.65	35.48	87.65	35.48	79.27
ESR	91.36	19.35	85.55	30.00	79.79
PCT	62.35	38.71	84.17	16.44	58.55

PPV, positive predictive value; NPV, negative predictive value; mNGS, metagenomic next-generation sequencing; CT, computed tomography; MRI, magnetic resonance imaging; CRP, c-reactive protein; ESR, erythrocyte sedimentation rate; PCT, procalcitonin.

## 4 Discussion

The early symptoms of SI, such as back pain, fever, and abnormal spinal morphology, are not specific, and the delay from the first symptom to the diagnosis is often 2 to 6 months (Babic and Simpfendorfer, 2017; Tsantes et al., 2020; Gregori et al., 2019). Although SI accounts for only 2% to 7% of the incidence of osteomyelitis throughout the body, failure to receive timely and accurate diagnosis and treatment can lead to severe consequences, including spinal deformities, neural function damage, paralysis, and even death (Srinivasan et al., 2014; Lener et al., 2018; Guo et al., 2022). Therefore, exploring new diagnostic methods to improve the accuracy of SI diagnosis is particularly important. mNGS has higher sensitivity than traditional culture techniques and shows great potential for identifying SI pathogens.

This study showed that the positivity rate of mNGS (77.78%) in the diagnosis of SI was significantly higher than that of microbial culture (27.16%), which is consistent with other reports (Guo et al., 2022). Among the 86 patients with SI and negative culture results, mNGS successfully detected the pathogens. In the diagnosis of complex polymicrobial infections, mNGS also outperformed traditional culture, which is consistent with a study by Mei et al (Mei et al., 2023). This reflects the high sensitivity of mNGS for detecting SI pathogens and its unique advantages in diagnosing complex infections. However, four culture-positive SI cases were missed by mNGS, suggesting that mNGS has limitations, especially with respect to the lack of a unified consensus on judgment criteria (Jiang et al., 2023; Greninger, 2018). Further investigation is required to determine whether the detected organisms are pathogenic. Among the 40 SI cases that were positive on both culture and mNGS, the results were highly consistent (77.50%). However, four cases were completely inconsistent, and after anti-infective treatment covering both pathogens, the prognosis was good; however, the specific source of infection remained unclear. Although this could have been due to multiple infections, the possibility of specimen contamination cannot be excluded. Therefore, strictly following sterilization and experimental protocols, ensuring the accurate use of blank controls, and promptly correcting false-positive results potentially caused by contamination are crucial (Tan et al., 2024).

In this study, we conducted an in-depth comparison of the differences between microbial cultures and mNGS for detecting pathogenic strains. Microbial cultures primarily contained *S. aureus, E. coli* and *B. melitensis*. In contrast, mNGS exhibited significant advantages in terms of the number and types of strains detected, with *S. aureus* and *M. tuberculosis* ranking in the top two positions, which is consistent with previous studies (Zhang et al., 2022, Zhang et al., 2023). Not only did mNGS detect common pathogenic microorganisms in SI, but it also identified difficult-to-culture or potentially opportunistic microorganisms such as *M. tuberculosis*, anaerobes, *Brucella*, and *C. burnetii*. These difficult-to-culture bacteria are difficult to detect in conventional cultures, even after extended culture times. Considering the high incidence of *M. tuberculosis* in China (Dong et al., 2023) and the commonality of *Brucella* infections in animal husbandry areas (Jiang and Kan, 2020), mNGS shows tremendous potential for identifying these difficult-to-culture infectious pathogens (Jin et al., 2023; Du et al., 2023; Yang et al., 2023). Furthermore, mNGS revealed that most *Streptococci* and anaerobes detected in patients with SI were normal oral microbiota. A report indicates that normal oral microbiota may enter the bloodstream in the context of oral diseases, becoming a significant factor in the occurrence of SI (Kilinc et al., 2024). Therefore, during clinical diagnosis and treatment, comprehensive judgment should be made by considering both the patient’s clinical manifestations and the possibility of specimen contamination. If the detected pathogenic microorganisms align with clinical expectations, the accuracy and specificity of sequencing results should be confirmed. When interpreting opportunistic pathogens, clinicians should exclude contamination and background microorganisms, taking into account the patient’s immune status and the consistency with clinical manifestations (Chinese Society of Laboratory Medicine, 2020). Regarding the detected *C. burnetii*, although it has rarely been reported in literature, clinicians should consider the possibility of Q fever when facing slowly progressive spinal cord disease with negative culture results (Lundy et al., 2019).

This study found no significant difference in the detection rates between the antibiotic-use and non-use groups using mNGS. This result reflects the advantage of mNGS for pathogen detection as it directly detects pathogenic nucleic acids and is not affected by antibiotic use during the detection process. This also suggests that in clinical settings, for patients who have undergone empirical treatment with antibiotics, the selection of mNGS is more conducive to identifying pathogens. Early identification of the pathogen, followed by the administration of targeted antibiotics and the reduction of unnecessary use of broad-spectrum antibiotics, is of great significance in improving patient prognosis and alleviating economic burdens. Selection of the sample type is crucial for improving the detection rate of pathogens. Surgical sampling significantly increased the detection rate of SI microbial cultures (from 11.43% to 31.50%), whereas the detection rate of mNGS showed no significant difference (from 80.00% to 77.17%). This reflects the flexibility of mNGS in sample type selection, which is almost unaffected by specimen type and can sensitively detect pathogens, even with small amounts of specimen (Duan et al., 2021). Needle biopsy is a viable option for patients who cannot or are unwilling to undergo surgical treatment and mNGS can detect pathogens in such patients in a better manner.

Currently, imaging techniques such as CT and MRI, as well as inflammatory markers such as CRP, ESR, and PCT, are commonly used in the diagnosis of SI (Jeong et al., 2015; Lener et al., 2018). Our study showed that ESR was the most sensitive diagnostic method for SI, with a sensitivity of 91.36%, while inflammatory markers, CT, and MRI were also relatively sensitive. However, the specificities of these indicator-based assays were not as high as that of mNGS. Additionally, these imaging- and inflammatory marker-based techniques cannot directly determine the type of pathogen, which limits their usefulness for treatment. Microbial culture plays an irreplaceable role in the diagnosis and treatment of SI and provides crucial information on antibiotic sensitivity. However, its sensitivity is only 27.16%, which may have led to missed diagnoses. In contrast, mNGS has a significantly higher sensitivity (77.78%) and better specificity than imaging and inflammatory markers, serving as an important complement to microbial culture and reducing the missed diagnosis rate of SI. Additionally, while microbial culture, imaging, and inflammatory markers currently still offer cost advantages, the ongoing advancements in mNGS are leading to a continuous reduction in its testing costs. A comprehensive mNGS analysis using imaging and laboratory indicators can improve the accuracy of SI diagnosis and provide precise treatment for patients.

The establishment of thresholds for pathogen diagnosis using mNGS is influenced by numerous factors, including sequencing platforms, sequencing protocols, specimen types, pathogen species, and patient conditions. Currently, there is a lack of universally accepted diagnostic thresholds (Expert Group on Consensus for High-Throughput Sequencing, 2023). Consequently, the detection of a small number of sequence reads in sterile specimens often poses a challenge in distinguishing between genuine infection and contamination. While increasing sequencing depth allows for the detection of a greater variety and quantity of pathogens, thereby enhancing the ability to identify low-abundance pathogens, it also results in an increase in both sequencing costs and analysis time (Zhang et al., 2019; Liu et al., 2022). Although this study has established positive criteria based on expert consensus and preliminary laboratory data, relying solely on relative abundance and sequence count for judgment may lead to false-positive or false-negative results. Therefore, when low sequence counts are detected for clinically significant and difficult-to-detect pathogens such as *M. tuberculosis* and *Brucella* species, a comprehensive multidisciplinary assessment incorporating the patient’s clinical information is necessary. Such assessments should be continually validated and refined in clinical practice.

This study had some limitations. ① Although the sample size was relatively large, the study was limited by the retrospective single-center study nature, which may have led to selection bias. Moreover, the high cost of mNGS technology restricts the sample size. ② The diagnostic criteria for SI have not yet been unified. Although this study used histopathology as a reference to calculate sensitivity and specificity, pathological results can be difficult to distinguish between inflammation and infection, potentially introducing bias into the results. ③ Considering the diversity of different sequencing platforms, sequencing workflows and the background bacteria of samples, there is still a lack of widely accepted and rigorously validated methods to ensure that mNGS meets the standards of test validation, reproducibility and quality assurance. ④ Due to technical limitations of clinical mNGS, sequencing costs, and database constraints, it is often difficult for mNGS to obtain the sequences of all microorganisms in a sample, which may lead to the omission of pathogenic microorganisms at low concentrations in clinical samples (Yin et al., 2024). Additionally, due to database biases and small sequence read length (50 bp) it is also possible for mNGS to yield false positives or misalignments between closely related organisms.

## 5 Conclusion

The pathogens causing SI are complex and diverse. In addition to *S. aureus* and *M. tuberculosis*, anaerobes, *Brucella*, *C. burnetii*, and other pathogens should receive clinical attention. The mNGS technique, which is not limited to antibiotics or sample types, significantly improves the detection rate of SI pathogens, especially those that are difficult to identify by microbial culture. Based on the application of mNGS, clinicians can gain a more comprehensive understanding of the etiological characteristics of SI, enabling the early determination of precise antibiotic treatment plans. This not only helps reduce the overuse of empirical antibiotics and improves treatment efficacy but also significantly improves patient outcomes and reduces unnecessary medical costs. It is recommended that in clinical practice, mNGS be combined with imaging and laboratory indicators for suspected SI cases to enhance the diagnostic accuracy and better serve patients.

## Data Availability

The original contributions presented in the study are publicly available. This data can be found here: NCBI: PRJNA1203379.
